# Catecholamines modulate pathogen-probiotic-host interactions: insights from *Pseudomonas aeruginosa* and *Pediococcus pentosaceus*

**DOI:** 10.3389/fmicb.2026.1833953

**Published:** 2026-07-17

**Authors:** Meryem Boujnane, Aurélie Budin-Verneuil, Héloïse Bizière-Maco, Salomé Lecoutour, Paloma López, Béatrice Labat, Nathalie Connil

**Affiliations:** 1 CBSA UR 4312, Univ Rouen Normandie, Normandy Univ, Rouen, France; 2 CBSA UR 4312, Univ Caen Normandie, Normandy Univ, Caen, France; 3 Departamento de Biotecnología, Centro de Investigaciones Biológicas Margarita Salas (CIB, CSIC), Madrid, Spain; 4 PBS UMR 6270, Univ Rouen Normandie, INSA Rouen Normandie, CNRS, Normandy Univ, Rouen, France

**Keywords:** bacteria-host interactions, catecholamines, probiotic, pathogen, *Pediococcus pentosaceus*, *Pseudomonas aeruginosa*

## Abstract

Stress-related catecholamine hormones, including epinephrine (Epi), norepinephrine (NE), and dopamine (Dopa), are increasingly recognized as modulators of bacterial physiology. However, the human microbiota represents a highly interconnected network of commensal and pathogenic species that continuously respond to host-derived and environmental cues, and how these signals influence interspecies interactions within microbial communities remains poorly understood. Here, we investigated the impact of catecholamines on the interaction between the pathogen *Pseudomonas aeruginosa* H103 and the probiotic *Pediococcus pentosaceus* MZF16. Catecholamine exposure increased the total biomass of dual-species biofilms while maintaining the predominance of MZF16. These hormones also modified bacterial co-aggregation and adhesion to abiotic surfaces and enhanced the anti-adhesion activity of MZF16 against *P. aeruginosa* H103 in human intestinal epithelial cells. Moreover, NE modulated the virulence of H103 in the presence of the probiotic strain, with distinct effects observed in human cells and in the *Galleria mellonella* infection model. Together, these findings demonstrate that catecholamines can modulate competitive interactions between commensal and pathogenic bacteria, suggesting that host neuroendocrine signals may contribute to shaping microbial community dynamics, regulating host–microbiota homeostasis, and influencing infection processes.

## Introduction

Catecholamines, commonly referred to as stress hormones, include epinephrine (Epi), norepinephrine (NE), and dopamine (Dopa). These molecules function both as neurotransmitters and endocrine mediators, exerting a wide range of effects on host physiology. Increasing evidence indicates that catecholamines can also influence the behavior of the resident microbiota (Lyte, 2014). Early studies in microbial endocrinology primarily focused on their ability to stimulate bacterial growth (Lyte and Ernst, 1992). More recent research has demonstrated that these hormones can also enhance microbial virulence (Freestone et al., 2008), highlighting a complex interplay between host stress signaling and bacterial pathogenicity (Boukerb et al., 2021; Luqman, 2023). The responsiveness of several pathogenic bacteria, including *Escherichia coli, Salmonella* Typhimurium, and *Pseudomonas aeruginosa*, to catecholamines is now well established (Bansal et al., 2007; Karavolos et al., 2008; Cambronel et al., 2019; Boujnane et al., 2026). Emerging evidence further suggests that commensal and probiotic bacteria may also perceive and respond to these host-derived signals (Cambronel et al., 2020). In addition, some gut-associated bacteria have been reported to produce catecholamine-like compounds; for example, strains of *Escherichia coli* and *Bacillus* spp. have been shown to synthesize dopamine, norepinephrine, or related catechol structures under specific conditions, supporting the existence of a bidirectional microbial contribution to the local intestinal catecholamine availability (Miri et al., 2023).

Microbial communities associated with the human body are continuously exposed to host-derived molecules and maintain complex networks of interspecies communication (Gannesen et al., 2024a). The interactions between host humoral factors and microbial populations have therefore become an important focus of microbial endocrinology (Ovcharova et al., 2021). Nevertheless, relatively few studies have examined how eukaryotic hormones, including natriuretic peptides, sex hormones, and catecholamines, influence bacterial community dynamics and host-microbe interactions (Gannesen et al., 2018; Mart'yanov et al., 2021; Ovcharova et al., 2021; Kiseleva et al., 2022; Diuvenji et al., 2023; Kalayci-Yüksek et al., 2021).

Polymicrobial infections are strongly shaped by cell-to-cell communication and environmental cues, and fluctuations in host-derived catecholamines during infection may therefore alter bacterial interactions and influence disease progression and persistence (Sarkodie et al., 2019). Moreover, clinical and experimental observations further support this relevance by linking elevated catecholamine levels to shifts in gut microbial composition, suggesting that host stress responses can reshape microbial ecosystems (Alcock et al., 2025). However, despite these advances, the impact of catecholamines on interactions between pathogenic and beneficial bacteria remains poorly understood. In this context, interactions between pathogenic and beneficial microorganisms provide a useful basis for exploring how host-derived signals influence microbial competition and behavior.

In the present study, we focused on the interaction between *P. aeruginosa* H103, a ubiquitous opportunistic pathogen frequently implicated in nosocomial infections, and *Pediococcus pentosaceus* MZF16, a Gram-positive lactic acid bacterium recognized for its probiotic potential (Zommiti et al., 2018). *P. aeruginosa* is characterized by its capacity to form robust biofilms, its intrinsic resistance to multiple antibiotics, and the presence of complex quorum-sensing (QS) networks that regulate numerous virulence factors (Moradali et al., 2017). In contrast, MZF16 is highly tolerant to gastrointestinal conditions and contributes to beneficial modulation of host-associated microbial communities. We previously demonstrated that this strain attenuates several QS-regulated phenotypes of *P. aeruginosa*, including biofilm formation, motility, and the production of key virulence factors, while effectively competing with the pathogen for intestinal colonization sites. These effects may involve mechanisms such as metabolic cross-talk, signal interference, or competition for environmental cues associated with QS regulation (Boujnane et al., 2025).

Although these microorganisms belong to distinct ecological groups, they may encounter each other in shared environments such as food matrices or transiently within the gastrointestinal tract, where competition for nutrients, ecological niches, and host adhesion sites may occur. Such coexistence provides opportunities for antagonistic or modulatory interactions that can influence pathogen colonization and virulence. This experimental pairing offers an appropriate setting to examine how host-derived hormones modulate competitive behaviors between pathogenic and probiotic bacteria. Accordingly, we evaluated the effects of Epi, NE, and Dopa on the interplay between *P. aeruginosa* H103 and *P. pentosaceus* MZF16.

## Material and methods

### Chemical molecules

Water-soluble catecholamines: Epi, NE, and Dopa, were prepared at 10 mM stock solutions in sterile distilled water, filtered through 0.22 μm membrane and stored at −20 °C until use. All molecules were tested at 100 μM as final concentration, previously used to mimic supraphysiological conditions (Freestone et al., 1999; Boujnane et al., 2026).

### Bacterial strains and culture conditions

Two bacterial strains were used in this study: the opportunistic pathogen *P. aeruginosa* H103 and the probiotic *Pediococcus pentosaceus* MZF16. They were respectively cultured at 37 °C under a rotary shaker (180 rpm) in Luria Bertani (LB) broth, and in static conditions in Man, Rogosa, and Sharpe (MRS) broth.

### Construction of *P. aeruginosa* H103-GFP and *Pediococcus pentosaceus* MZF16-mCherry

The construction of fluorescent bacterial strains was described in our previous work (Boujnane et al., 2025). Briefly, *P. aeruginosa* H103 was grown in LB medium until reaching an OD_600nm_ of 0.5, then harvested by centrifugation at 7000 rpm for 10 min at 4 °C. Electrocompetent *P. aeruginosa* cells were prepared and transformed with 50 ng of plasmid pHC60 (carrying the *gfp* and *tetM* genes encoding the green fluorescent protein (GFP) and the tetracycline efflux protein, respectively) by electroporation at 1.8 kV in a 0.2 cm cuvette. After pulsing, 1 mL of LB medium was immediately added, and the bacteria were incubated at 37 °C for 2 h in a rotary shaker (180 rpm). *P. aeruginosa* H103-GFP transformants were selected for tetracycline resistance on LB agar supplemented with tetracycline (100 μg/mL).

For *Pediococcus pentosaceus* MZF16, cultures were grown in MRS medium to an OD_600nm_ of 0.8 (approximatively 1 × 10^8^ CFU/mL), harvested by centrifugation at 7,000 rpm for 10 min at 4 °C, washed twice with phosphate-buffered saline (PBS, pH 7.2), and resuspended in 1 mL of lysozyme solution (2,000 U/mL). The suspension was incubated for 20 min at 37 °C to weaken the cell wall. Electrocompetent cells were then transformed with 0.5 μg of the broad host range pRCR12 plasmid, carrying the *cat* and the *mrfp* genes, which encode, respectively, the chloramphenicol acetyl transferase and the monomeric red fluorescent protein *mCherry* (Russo et al., 2015) by electroporation at 2.5 kV in a 0.2-cm cuvette. After electroporation, 500 μL of MRS medium was immediately added to the cuvette, and the cells were incubated at 37 °C for 2 h. Transformants (*Pediococcus pentosaceus* MZF16-mCherry) were selected for chloramphenicol resistance on MRS-agar supplemented with chloramphenicol (100 μg/mL).

### Dual-species biofilm formation on glass surface

Dual-species biofilm formation on glass surfaces was assessed using the fluorescent strains *P. aeruginosa* H103-GFP and *Pediococcus pentosaceus* MZF16-mCherry. Overnight cultures were centrifuged (8,000 rpm, 5 min) and resuspended in Dulbecco's Modified Eagle Medium (DMEM) supplemented with 20% heat-inactivated fetal bovine serum (DMEM-FBS 20%). Suspensions of *P. aeruginosa* H103-GFP alone or mixed with *Pediococcus pentosaceus* MZF16-mCherry (1:100 ratio) were prepared in the presence or absence of the catecholamines Epi, NE, and Dopa (100 μM), then incubated in 24-well Sensoplates (Greiner) for 24 h at 37 °C under static conditions. After incubation, the medium was removed and wells were gently washed three times with sterile PBS to eliminate non-adherent cells. Biofilms were analyzed by confocal laser scanning microscopy (CLSM) as described below.

Biofilm imaging was performed using a Zeiss LSM710 confocal microscope equipped with a 40 × oil-immersion objective. GFP-labeled *P. aeruginosa* was visualized using 488-nm excitation and 550-nm emission detection, whereas mCherry-labeled *Pediococcus pentosaceus* was detected using 514-nm excitation and 540–590-nm emission. Z-stacks were acquired at 1-μm intervals throughout the entire biofilm depth and reconstructed in 3D using Zen 2.1 software (Zeiss). Quantitative image analysis was performed with COMSTAT (Heydorn et al., 2000; Vorregaard, 2008) to determine bacterial biovolume and mean biofilm thickness.

### Bacterial aggregation and adhesion to glass surface

Mixed suspensions of *P. aeruginosa* H103 and *Pediococcus pentosaceus* MZF16 were prepared in PBS and adjusted to an OD600 of 0.3. Catecholamines (Epi, NE, or Dopa) were then added or not to the suspensions depending on the conditions tested. Microscope coverslips (20 mm) were placed in Petri dishes, and the bacterial suspensions were poured onto them and incubated for 2 h at room temperature. After incubation, coverslips were gently washed twice with PBS, fixed with 2.5% glutaraldehyde for 1 h 30 min, and washed three additional times. Samples were then dehydrated through a graded ethanol series (50% to 100%, 10% increments, 15 min per step). Dried coverslips were mounted on aluminum stubs and sputter-coated with gold using a 108 Auto Sputter Coater (Ted Pella, USA). Co-aggregation and bacterial adhesion were examined by scanning electron microscopy (SEM) using a JCM-6000 microscope (JEOL, Japan).

### Caco-2/TC7 cell culture

Human colon adenocarcinoma Caco-2/TC7 cells (Chantret et al., 1994) were cultured in Dulbecco's Modified Eagle Medium (DMEM; Lonza, Basel, Switzerland) supplemented with 20% heat-inactivated fetal bovine serum (FBS; Sigma-Aldrich, USA) and penicillin–streptomycin (100 μg/mL). Cells were maintained at 37 °C in a humidified 5% CO_2_ atmosphere, and the culture medium was renewed every 2 days. Cells were passaged at approximately 90% confluence. For adhesion and cytotoxicity assays, approximately 10^4^ cells/mL were seeded into 24-well tissue culture-treated plates and grown to confluence.

To assess bacterial adhesion and cytotoxicity, suspensions of *P. aeruginosa* H103 (10^6^ CFU/mL) alone or co-cultured with *Pediococcus pentosaceus* MZF16 (10^8^ CFU/mL) were prepared in DMEM lacking FBS and antibiotics, with or without catecholamines (Epi, NE, Dopa). The 1:100 H103:MZF16 ratio was selected based on our previous findings (Boujnane et al., 2025).

### Bacterial adhesion to Caco-2/TC7 cells

To evaluate bacterial adhesion by competitive exclusion, bacterial suspensions were added to confluent Caco-2/TC7 monolayers and incubated for 3 h at 37 °C in a humidified atmosphere containing 5% CO_2_. After incubation, monolayers were washed twice with sterile PBS to remove non-adherent bacteria and lysed with 0.1% Triton X-100. The cell lysates were serially diluted and plated on Cetrimide agar, selective medium for enumeration of adherent *P. aeruginosa* H103 and MRS agar for *P. pentosaceus* MZF16. Plates were incubated at 37 °C, and adherent bacteria were enumerated.

### Cytotoxicity assay

The cytotoxicity induced by *P. aeruginosa* H103 on Caco-2/TC7 monolayers was evaluated in the presence or absence of *Pediococcus pentosaceus* MZF16 and NE, the predominant catecholamine in the human gastrointestinal tract (Lyte, 2014). Cells monolayers were exposed for 16 h to bacterial suspensions containing *P. aeruginosa* H103 alone or coculture with *P. pentosaceus* MZF16 at a ratio of 1:100 (H103:MZF16). Following overnight incubation, culture supernatants were collected, and cytotoxicity was assessed by quantifying lactate dehydrogenase (LDH) release using the CyQUANT™ LDH Cytotoxicity Assay Kit (Invitrogen), following the manufacturer's instructions.

### *Galleria mellonella* infection model

The virulence of *P. aeruginosa* H103 in the presence or absence of *Pediococcus pentosaceus* MZF16 and the catecholamine NE was assessed using the *Galleria mellonella* infection model as previously described (Coupri et al., 2021), with minor modifications. Overnight cultures of *P. aeruginosa* H103 and *Pediococcus pentosaceus* MZF16 were grown in LB and MRS broths, respectively, harvested by centrifugation, washed in sterile physiological water, and resuspended to prepare mixed inocula. Final suspensions were adjusted to approximately 10^4^CFU/mL for H103 and 10^6^CFU/mL for MZF16 (1:100 ratio), with or without the addition of NE.

Six-week-old larvae were injected with 10 μL of the bacterial suspension into the last left proleg using a syringe pump (Thermo Fisher Scientific, USA) fitted with a 26G needle (Terumo, Japan). Following injection, larvae were transferred to sterile Petri dishes and incubated at 37 °C. Survival was monitored every 2 h for 24 h post-infection. Three independent experiments were performed, totaling at least 90 larvae per condition. Survival data were analyzed and visualized using Kaplan–Meier curves.

### Statistical analysis

All statistical analyses were performed using GraphPad Prism. Depending on the dataset, unpaired two-sample tests, one-way ANOVA, or multiple-comparison tests were applied. Larval survival curves were analyzed using the log-rank (Mantel–Cox) test. All experiments were performed at least in triplicate, and statistical significance was set at *p* < 0.05.

## Results

### Catecholamines increase dual-species biofilm formation

**Figure 1:**
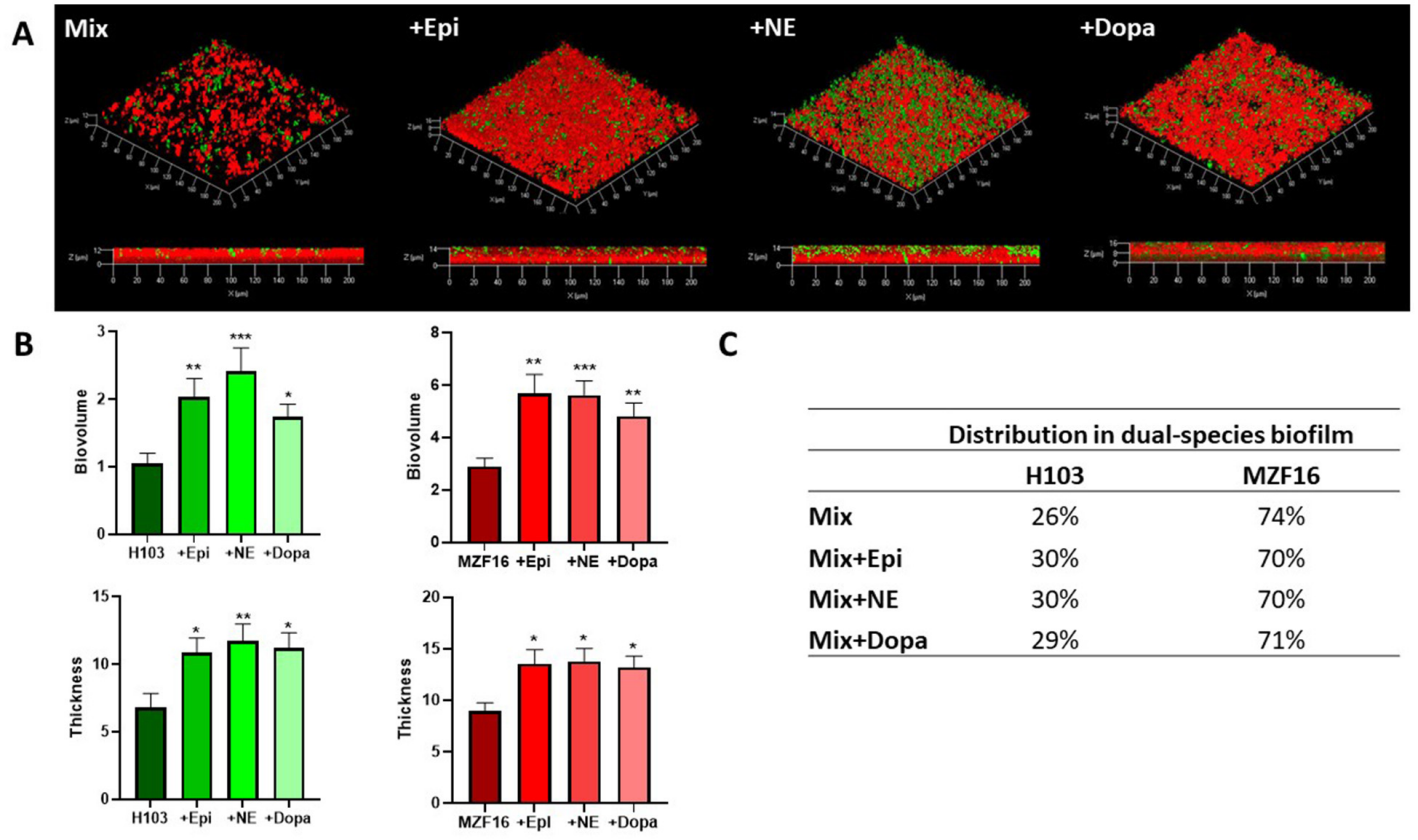
Effect of catecholamines on dual-species biofilm formation by *Pseudomonas aeruginosa* H103-GFP and *Pediococcus pentosaceus* MZF16-mCherry. **(A)** CLSM images of 24 h biofilms. **(B)** COMSTAT quantification of biovolume and mean thickness for each strain. **(C)** Percentage distribution of both strains within the biofilm. *p < 0.05, **p < 0.01, ***p < 0.001. A non-parametric ANOVA test was used.

Dual-species biofilms formed by *Pseudomonas aeruginosa* H103-GFP and *Pediococcus pentosaceus* MZF16-mCherry were assessed on glass surfaces in the presence or absence of the catecholamines Epi, NE, and Dopa. Confocal microscopy showed an overall increase in biofilm development upon hormone addition (Figure 1A). Quantification of biovolume and thickness for each individual strain within the mixed biofilm revealed a significant, approximately twofold rise (Figure 1B). However, catecholamines did not substantially modify strain distribution within the dual-species biofilm, and *Pediococcus pentosaceus* MZF16 remained predominant in all conditions (Figure 1C).

### Catecholamines alter bacterial co-aggregation and adhesion to glass surfaces

To examine the effect of catecholamines on bacterial co-aggregation and adhesion, co-cultures of *P. aeruginosa* H103 and *Pediococcus pentosaceus* MZF16 were treated with Epi, NE, or Dopa, and adhesion was assessed on glass coverslips. Samples were subsequently analyzed by scanning electron microscopy (SEM).

**Figure 2:**
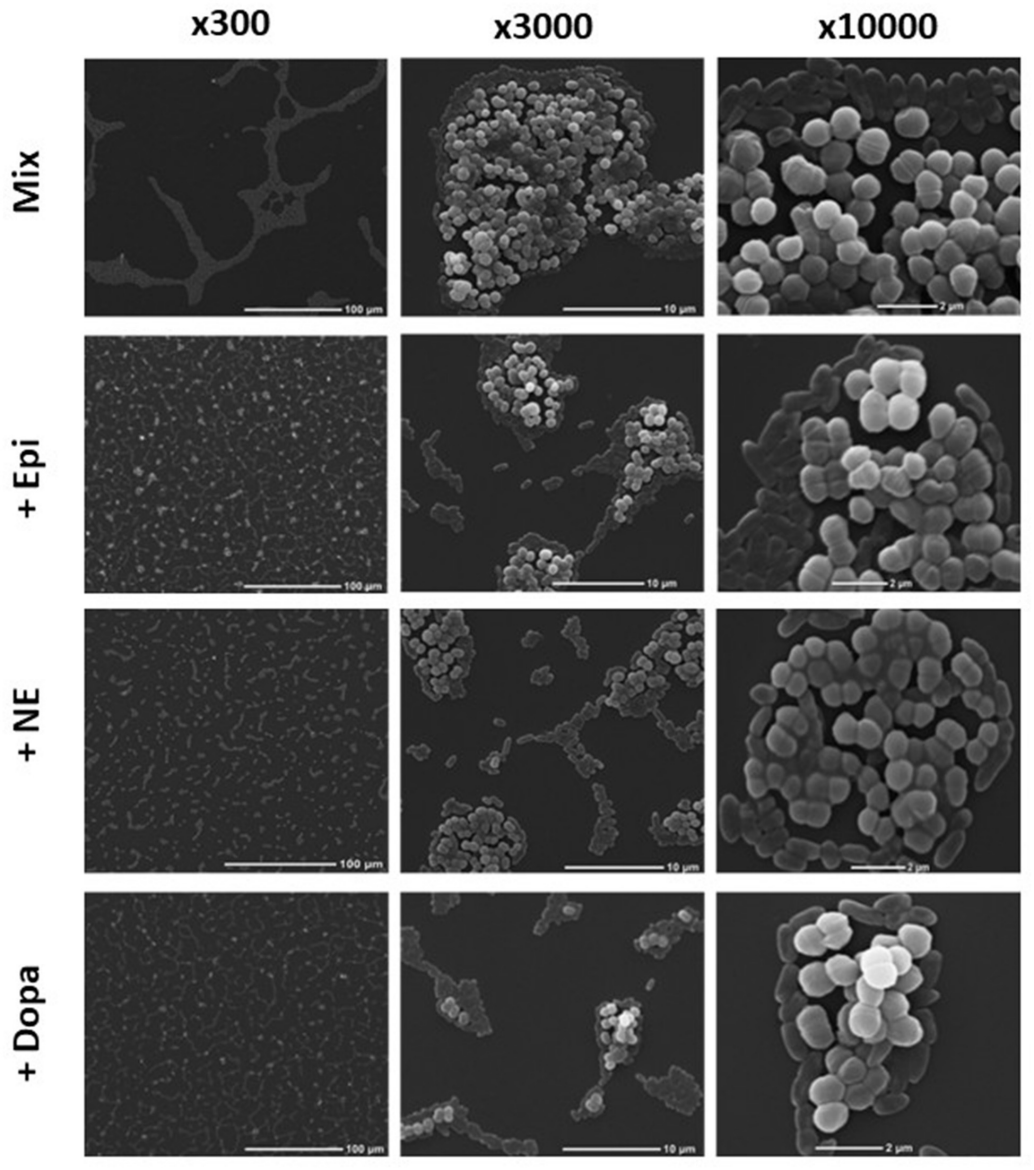
SEM analysis of bacterial co-aggregation and adhesion on glass surfaces at *t* = 2 h. Co-aggregation between *Pseudomonas aeruginosa* H103 and *Pediococcus pentosaceus* MZF16 was assessed in the presence or absence of the indicated catecholamines. Representative SEM images for each condition were acquired at 300×, 3,000×, and 10,000× magnifications.

Pronounced co-aggregation was observed between H103 and MZF16, forming compact and structured networks in absence of catecholamine (Figure 2, Mix, left panel). In contrast, catecholamine treatment (Epi, NE or Dopa) disrupted this organization, resulting in more dispersed and loosely associated aggregates. Co-aggregates also appeared less cohesive and visually less abundant following catecholamine exposure (Figure 2, middle panel). Interestingly, MZF16 aggregates were consistently observed on top of H103 clusters, suggesting a preferential spatial arrangement, and were not observed adhering to the surface independently; their abundance decreased notably in the presence of Dopa.

Overall, these observations indicate that catecholamines influence the structural organization of the co-aggregates formed by H103 and MZF16 on the glass surface.

### Catecholamines potentiate the anti-adhesion effect of *Pediococcus pentosaceus* MZF16 against *Pseudomonas aeruginosa* H103

**Figure 3:**
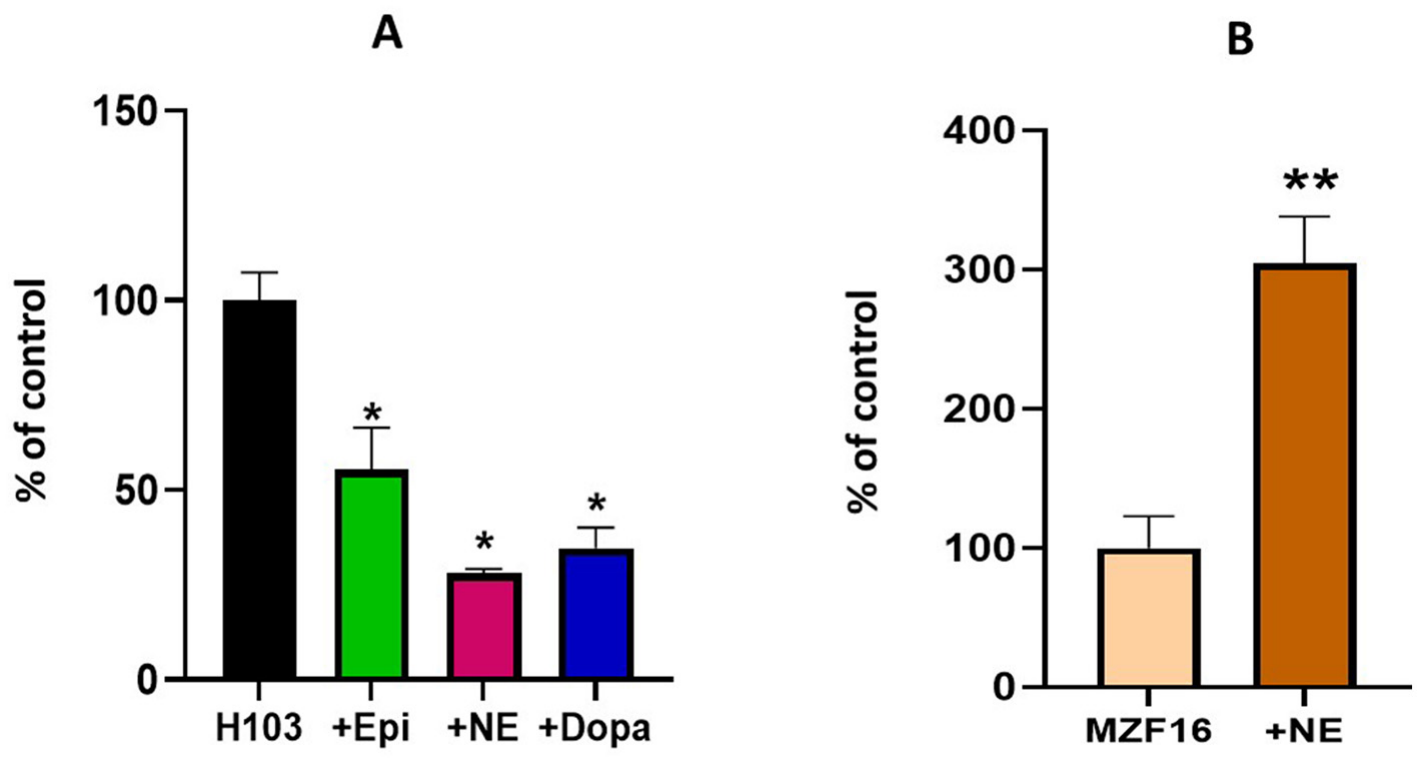
Effect of catecholamines on bacterial adhesion to Caco-2/TC7 cells. **(A)** Adhesion of *Pseudomonas aeruginosa* H103 to Caco-2/TC7 cells after 3 h incubation with mixed H103-MZF16 cultures (1:100) in the presence or absence of catecholamines (Epi, NE, or Dopa at 100 μM). Results are expressed relative to untreated co-cultures. **(B)** Effect of NE on *Pediococcus pentosaceus* MZF16 adhesion to Caco-2/TC7 cells compared to untreated MZF16. Statistical significance was determined using t-tests (*p < 0.05, **p < 0.01).

To investigate whether catecholamines influence bacterial adhesion to Caco-2/TC7 intestinal cells, a competitive exclusion assay was performed. We previously reported that MZF16 markedly reduces the adhesion of H103 to Caco-2/TC7 cells (Boujnane et al., 2025). Here, treatment with Epi, NE, or Dopa further enhanced the anti-adhesion activity of *Pediococcus pentosaceus* MZF16 against *P. aeruginosa* H103 by 45%, 72%, and 65%, respectively (Figure 3A). The pronounced anti-H103 adhesion effect observed for NE was likely due, at least in part, to NE-enhanced adhesion of the probiotic MZF16 itself (Figure 3B).

### Norepinephrine modulates *Pseudomonas aeruginosa* H103 virulence in the presence of *Pediococcus pentosaceus* MZF16

**Figure 4:**
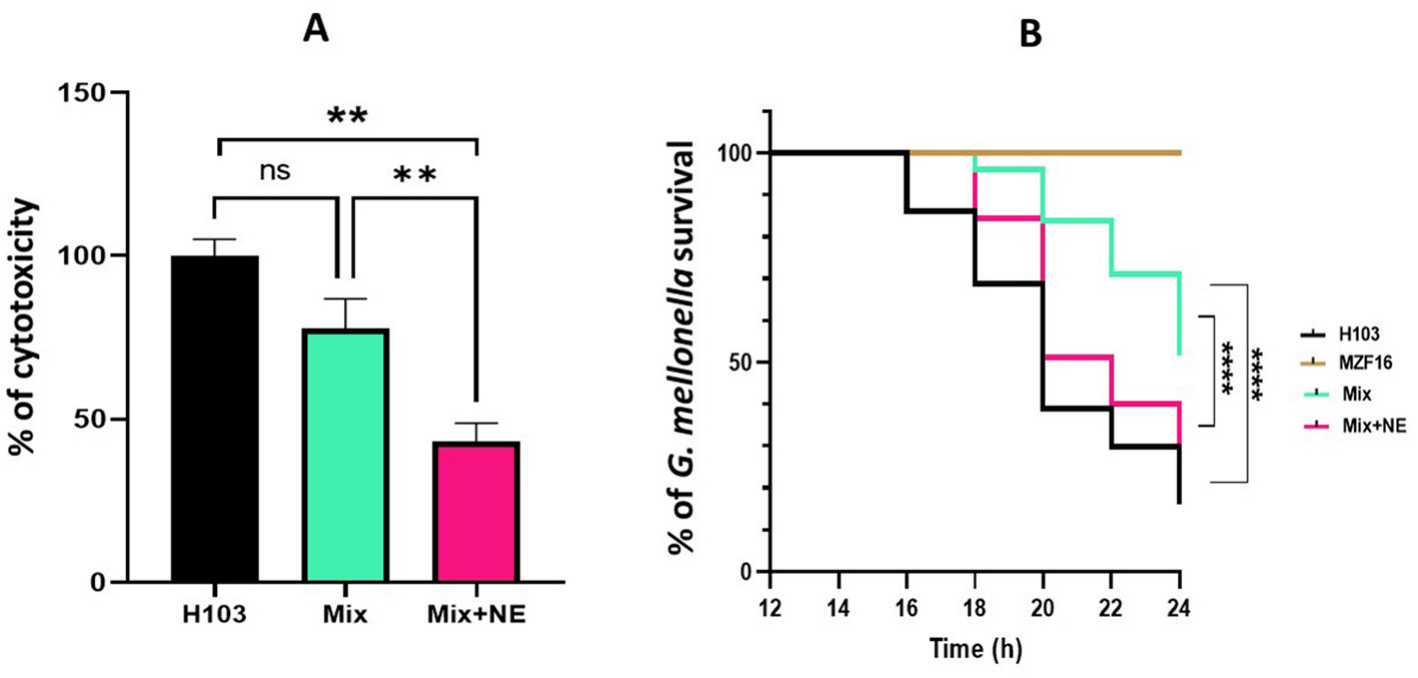
Effect of norepinephrine on *Pseudomonas aeruginosa* H103 cytotoxicity toward Caco-2/TC7 cells and on *in vivo* virulence in *Galleria mellonella*, during co-exposure with the probiotic *Pediococcus pentosaceus* MZF16. **(A)** Cytotoxicity of H103 toward Caco-2/TC7 cells co-cultured with MZF16 in the presence or absence of NE, assessed by LDH release after overnight incubation. **(B)** Survival of *Galleria mellonella* larvae following infection with H103 and MZF16 co-cultures pretreated with NE or left untreated for 24 h. **p < 0.01, ****p < 0.0001.

The cytotoxic activity of *P. aeruginosa* H103 was assessed on Caco-2/TC7 monolayers in the presence or absence of *Pediococcus pentosaceus* MZF16 and NE, the predominant catecholamine in the human gastrointestinal tract (Lyte, 2014). The results shown in Figure 4A indicate that the presence of *Pediococcus pentosaceus* MZF16 in the co-treatment mixture (Mix) slightly reduced the cytotoxicity induced by *P. aeruginosa* H103 on Caco-2/TC7 cells. This protective effect was markedly strengthened in the presence of NE, suggesting that this hormone may potentiate the probiotic's anti-pathogenic activity.

To explore how NE influences the *in vivo* interaction between *P. aeruginosa* H103 and *Pediococcus pentosaceus* MZF16, *Galleria mellonella* larvae were injected with individual strains and co-cultures that were either exposed or not exposed to this catecholamine. Figure 4B shows that the probiotic MZF16 significantly prolonged the lifespan of *G. mellonella* larvae infected with *P. aeruginosa* H103 (Mix compared to H103, *p* < 0.001), consistent with previous findings in a *C. elegans* model (Boujnane et al., 2025). Nevertheless, this protective effect of MZF16 on larvae infected by H103 was nearly completely abolished in the presence of NE (Mix+NE compared to Mix, *p* < 0.001), with a reduction of *G. mellonella* survival by approximately 58%. These results indicate that, in this animal model and under these conditions, the presence of catecholamines can favor the pathogen H103.

## Discussion

Epinephrine (Epi), norepinephrine (NE), and dopamine (Dopa) are catecholamines traditionally recognized for their roles as neurotransmitters and stress-related hormones in the host. Beyond these neurophysiological functions, increasing evidence indicates that catecholamines act as interkingdom signaling molecules capable of modulating bacterial physiology across diverse species, influencing growth, virulence, and the expression of associated genes (Boukerb et al., 2021; Boujnane et al., 2024).

Most previous studies have examined the effects of catecholamines on single bacterial species. However, *in vivo*, host-associated bacteria form complex communities where interspecies interactions strongly influence community structure and function. Whether catecholamines can modulate these interactions or alter community balance remains largely unknown. Thus, we investigated how catecholamines affect the interaction between the opportunistic pathogen *Pseudomonas aeruginosa* H103 and the probiotic strain *Pediococcus pentosaceus* MZF16. These two microorganisms were selected because they represent contrasting ecological and functional groups, allowing us to explore how host-derived hormones such as Epi, NE, and Dopa may influence pathogen-probiotic interactions.

Independent exposure to each of the three catecholamines significantly increased total biofilm biomass and average thickness on glass surfaces, in parallel with enhanced biofilm formation by each strain individually. Despite this overall increase, catecholamine treatment did not modify the relative distribution of the two species within mixed biofilms, with *Pediococcus pentosaceus* MZF16 remaining predominant. These findings indicate that catecholamines can stimulate dual-species biofilm formation without disrupting the established interspecies balance, suggesting a potential role in modulating microbial community homeostasis in our experimental conditions.

In contrast, other studies have reported hormone-dependent shifts in interspecies competition. For example, NE was shown to accelerate the suppression of *Staphylococcus aureus* by *S. epidermidis* in dual-species biofilms (Mart'yanov et al., 2021).

Similarly, the effects of other host-derived signaling molecules, such as steroid hormones and natriuretic peptides, on biofilm formation and microbial dynamics are still poorly understood. For instance, natriuretic peptides (ANP, BNP, and CNP) have been shown to modulate dual-species biofilms. Indeed, in binary biofilms of *S. epidermidis* and *Cutibacterium acnes, S. epidermidis* initially predominates in CFU counts (Gannesen et al., 2024b). However, Atrial Natriuretic Peptide (ANP) exposure markedly shifts this balance by suppressing *S. epidermidis* growth while promoting *C. acnes* proliferation, resulting in a substantial increase in the relative abundance of *C. acnes* (Ovcharova et al., 2021). CNP alone minimally affects *C. acnes*, but in mixed biofilms it enhances *C. acnes* competitiveness and metabolic activity while predominantly inhibiting *S. epidermidis* (Ovcharova et al., 2023). Similarly, the steroid hormone estradiol shifts from inhibiting *Lactobacillus paracasei* in monoculture to stimulate it in dual-species biofilms with *Micrococcus luteus*, underscoring the critical role of microbial community context in modulating hormone responses (Kiseleva et al., 2022). These observations suggest that multiple host hormonal signals may act in concert or differentially to shape microbial community structure and interspecies competition.

Similarly, host-derived molecules such as natriuretic peptides or steroid hormones can markedly reshape microbial interactions, sometimes reversing competitive outcomes (Ovcharova et al., 2021, 2023; Kiseleva et al., 2022). Together, these observations highlight that host hormonal signals may differentially influence microbial community structure depending on species composition and interaction context.

In our study, we also examined bacterial co-aggregation and adhesion to glass surfaces under catecholamine exposure. Scanning electron microscopy showed that *P. aeruginosa* H103 and *Pediococcus pentosaceus* MZF16 normally form a dense, interconnected network, which became noticeably disrupted following treatment with Epi, NE, or Dopa. These observations suggest that host-derived catecholamines can alter the physical organization and surface attachment of mixed-species communities, potentially reshaping biofilm architecture and interspecies interactions.

In agreement with our findings, the co-aggregation and surface adhesion of *S. aureus* 209P and *Kytococcus schroeteri* H01 were altered in the presence of ANP hormone, due to a reduction in hydrophobic interactions and adhesion potential (Diuvenji et al., 2022). Conversely, ANP was associated with an increase in co-aggregates formation between *C. acnes* and *S. epidermidis*, along with changes in aggregate size relative to single cells (Ovcharova et al., 2021). Together, these findings indicate that eukaryotic hormones can modulate bacterial aggregation behavior in a species-specific manner. The mechanisms underlying these modulations remain largely unexplored.

Adhesion patterns observed on abiotic surfaces do not fully reflect the physiological conditions of the human intestine. To better approximate host-associated environments, we examined how catecholamines influence bacterial adhesion to intestinal epithelial Caco-2/TC7 cells. Our results show that catecholamines enhance the anti-adhesive activity of *Pediococcus pentosaceus* MZF16 against *P. aeruginosa* H103, suggesting that these hormones may modulate competition for epithelial attachment sites. However, such *in vitro* models remain simplified systems and cannot fully reproduce the complexity of the gut environment, including mucus layers, immune factors, and interactions with the resident microbiota.

Conversely, Niu et al. (2025) reported that NE attenuated several probiotic properties of *Levilactobacillus brevis*, including its inhibitory effect on the adhesion of the pathogenic *E. coli*. Moreover, NE reduced the strain protective histological and anti-inflammatory effects, as well as its ability to prevent *E. coli* colonization in an *in vivo* mouse model. In addition, NE weakened the anti-virulence activity of *L. brevis* against *E. coli, S. aureus*, and *P. aeruginosa* (Niu et al., 2025). Similarly, in our *G. mellonella in vivo* model, exposure to NE diminished the protective role of MZF16 against H103 virulence, as evidenced by decreased larval survival, but not the colonization rate (data not shown). These findings indicate that, under stress-mimicking conditions, catecholamines can compromise the beneficial *in vivo* anti-virulence properties of MZF16, highlighting the importance of considering host-derived hormonal signals when evaluating probiotic–pathogen interactions.

## Conclusion

Our findings highlight the need to further investigate how host-derived hormonal signals influence microbial homeostasis. The observed effects of catecholamines on *Pseudomonas aeruginosa* and *Pediococcus pentosaceus* show that host hormones can modulate not only individual bacterial behavior but also interspecies interactions, biofilm architecture, adhesion dynamics, and competitive outcomes. These results suggest that microbial endocrinology may play an important role in shaping microbial community composition, influencing pathogen colonization, and affecting the efficacy of probiotic interventions. Future studies integrating more physiologically relevant systems including host tissues, immune components, and complex microbiota will be essential to elucidate the mechanisms through which hormones regulate microbial behavior. A deeper understanding of these processes could open new avenues for modulating microbiota-host interactions for therapeutic or preventive purposes, particularly in contexts where stress or hormonal fluctuations alter microbial balance and infection risk.

## Data Availability

The raw data supporting the conclusions of this article will be made available by the authors, without undue reservation.

## References

[B1] Alcock J. Lin D. Setty P. Brown L.K. Dichosa A.E.K. Burnett B.J. . (2025). Catecholamine exposure and the gut microbiota in obstructive sleep apnea. PeerJ 13:e19203. doi: 10.7717/peerj.19203 40247843 PMC12005174

[B2] Bansal T. Englert D. Lee J. Hegde M. Wood T.K. Jayaraman A. (2007). Differential effects of epinephrine, norepinephrine, and indole on *Escherichia coli* O157:H7 chemotaxis, colonization, and gene expression. Infect. Immun. 75, 4597–4607. doi: 10.1128/IAI.00630-07 17591798 PMC1951185

[B3] Boujnane M. André G. Jeannot K. Boukerb A.M. Connil N. (2026). A QseBC-like system is involved in motility and biofilm formation responses to catecholamines in *Pseudomonas aeruginosa* PAO1. Microb. Pathog. 210:108162. doi: 10.1016/j.micpath.2025.108162 41203086

[B4] Boujnane M. Boukerb A.M. Connil N. (2024). Bacterial gene expression in response to catecholamine stress hormones. Curr. Opin. Endocr. Metab. Res. 36:100543. doi: 10.1016/j.coemr.2024.100543

[B5] Boujnane M. Zommiti M. Lesouhaitier O. Ferchichi M. Tahrioui A. Boukerb A.M. . (2025). *Pediococcus pentosaceus* MZF16 Probiotic Strain Prevents In Vitro Cytotoxic Effects of *Pseudomonas aeruginosa* H103 and Prolongs the Lifespan of *Caenorhabditis elegans*. Pathogens 14:244. doi: 10.3390/pathogens14030244 40137729 PMC11945076

[B6] Boukerb A.M. Cambronel M. Rodrigues S. Mesguida O. Knowlton R. Feuilloley M.G.J. . (2021). Inter-Kingdom Signaling of Stress Hormones: Sensing, Transport and Modulation of Bacterial Physiology. Front. Microbiol. 12:690942. doi: 10.3389/fmicb.2021.690942 34690943 PMC8526972

[B7] Cambronel M. Nilly F. Mesguida O. Boukerb A.M. Racine P.-J. Baccouri O. . (2020). Influence of Catecholamines (Epinephrine/Norepinephrine) on Biofilm Formation and Adhesion in Pathogenic and Probiotic Strains of *Enterococcus faecalis*. Front. Microbiol. 11:1501. doi: 10.3389/fmicb.2020.01501 32849320 PMC7396564

[B8] Cambronel M. Tortuel D. Biaggini K. Maillot O. Taupin L. Réhel K. . (2019). Epinephrine affects motility, and increases adhesion, biofilm and virulence of *Pseudomonas aeruginosa* H103. Sci. Rep. 9:20203. doi: 10.1038/s41598-019-56666-7 31882963 PMC6934790

[B9] Chantret I. Rodolosse A. Barbat A. Dussaulx E. Brot-Laroche E Zweibaum A. . (1994). Differential Expression of Sucrase-Isomaltase in Clones Isolated from Early and Late Passages of the Cell Line Caco-2: Evidence for Glucose-Dependent Negative Regulation. J. Cell Sci. 107, 213–225. doi: 10.1242/jcs.107.1.213 8175910

[B10] Coupri D. Verneuil N. Hartke A. Liebaut A. Lequeux T. Pfund E. . (2021). Inhibition of d-alanylation of teichoic acids overcomes resistance of methicillin-resistant *Staphylococcus aureus*. J. Antimicrob. Chemother. 76, 2778–2786. doi: 10.1093/jac/dkab287 34450626 PMC8521394

[B11] Diuvenji E.V. Nevolina E.D. Mart'yanov S.V. Zhurina M.A. Kalmantaeva O.V. Makarova M.A. . (2022). Binary Biofilms of *Staphylococcus aureus* 209P and *Kytococcus schroeteri* H01: Dualistic Role of Kytococci and Cell Adhesion Alterations in the Presence of the A-Type Natriuretic Peptide. Microbiol. 91, 563–576. doi: 10.1134/S002626172260118X

[B12] Diuvenji E.V. Nevolina E.D. Solovyev I.D. Sukhacheva M.V. Mart'yanov S.V. Novikova A.S. . (2023). A-Type Natriuretic Peptide Alters the Impact of Azithromycin on Planktonic Culture and on (Monospecies and Binary) Biofilms of Skin Bacteria *Kytococcus schroeteri* and *Staphylococcus aureus*. Microorganisms 11:2965. doi: 10.3390/microorganisms11122965 38138110 PMC10746058

[B13] Freestone P.P.E. Haigh R.D. Lyte M. (2008). Catecholamine inotrope resuscitation of antibiotic-damaged staphylococci and its blockade by specific receptor antagonists. J. Infect. Dis. 197, 1044–1052. doi: 10.1086/529202 18419472

[B14] Freestone P. P. Haigh R. D. Williams P. H. Lyte M. (1999). Stimulation of bacterial growth by heat-stable, norepinephrine-induced autoinducers. FEMS Microbiol. Lett. 172, 53–60. doi: 10.1111/j.1574-6968.1999.tb13449.x 10079527

[B15] Gannesen A.V. Lesouhaitier O. Racine P.-J. Barreau M. Netrusov A.I. Plakunov V.K. . (2018). Regulation of Monospecies and Mixed Biofilms Formation of Skin *Staphylococcus aureus* and *Cutibacterium acnes* by Human Natriuretic Peptides. Front. Microbiol. 9:2912. doi: 10.3389/fmicb.2018.02912 30619105 PMC6296281

[B16] Gannesen A.V. Ziganshin R.H. Ovcharova M.A. Mosolova A.M. Loginova N.A. Diuvenji E.V. . (2024b). Changes in the Protein Profiles of *Staphylococcus epidermidis* Planktonic Cultures and Biofilms under Anoxic Conditions in the Presence of the CNP Hormone. Microbiol. 93, 799–811. doi: 10.1134/S0026261724607188

[B17] Gannesen A. V. Mart'yanov S.V. Plakunov V.K. (2024a). How human hormones regulate human microbiota: Where are we in the middle of this terra incognita? Curr. Opin. Endocr. Metab. Res. 36:100537. doi: 10.1016/j.coemr.2024.100537

[B18] Heydorn A. Nielsen A.T. Hentzer M. Sternberg C. Givskov M. Ersbøll B.K. . (2000). Quantification of biofilm structures by the novel computer program COMSTAT. Microbiol. 146, 2395–2407. doi: 10.1099/00221287-146-10-2395 11021916

[B19] Kalayci-Yüksek F. Gümüş D. Ang-Küçüker M. (2021). Hormones Can Influence Antibiotic Susceptibilities Even in Mono- and Co-Culture Conditions. Acta Biol. Marisiensis 4, 39–49. doi: 10.2478/abmj-2021-0012

[B20] Karavolos M. H. Spencer H. Bulmer D. M. Thompson A. Winzer K. Williams P. . (2008). Adrenaline modulates the global transcriptional profile of Salmonella revealing a role in the antimicrobial peptide and oxidative stress resistance responses. BMC Genomics 9, 458. doi: 10.1186/1471-2164-9-458 18837991 PMC2576261

[B21] Kiseleva A.A. Solovyeva T.V. Ovcharova M.A. Geras'kina O.V. Mart'yanov S.V. Cherdyntseva T.A. (2022). Effect of β-Estradiol on Mono- and Mixed-Species Biofilms of Human Commensal Bacteria *Lactobacillus paracasei* AK508 and *Micrococcus luteus* C01 on Different Model Surfaces. Coatings 12:436. doi: 10.3390/coatings12040436

[B22] Luqman A. (2023). The orchestra of human bacteriome by hormones. Microb. Pathog. 180:106125. doi: 10.1016/j.micpath.2023.106125 37119938

[B23] Lyte M. (2014). Microbial endocrinology: Host-microbiota neuroendocrine interactions influencing brain and behavior. Gut Microbes 5, 381–389. doi: 10.4161/gmic.28682 24690573 PMC4153777

[B24] Lyte M. Ernst S. (1992). Catecholamine induced growth of gram negative bacteria. Life Sci. 50, 203–212. doi: 10.1016/0024-3205(92)90273-R 1731173

[B25] Mart'yanov S.V. Botchkova E.A. Plakunov V.K. Gannesen A.V. (2021). The Impact of Norepinephrine on Mono-Species and Dual-Species Staphylococcal Biofilms. Microorganisms 9:820. doi: 10.3390/microorganisms9040820 33924447 PMC8070549

[B26] Miri S. Yeo J. Abubaker S. Hammami R. (2023). Neuromicrobiology, an emerging neurometabolic facet of the gut microbiome? Front. Microbiol. 14, 1098412. doi: 10.3389/fmicb.2023.1098412 PMC9886687 36733917

[B27] Moradali M.F. Ghods S. Rehm B.H.A. (2017). *Pseudomonas aeruginosa* Lifestyle: A Paradigm for Adaptation, Survival, and Persistence. Front. Cell. Infect. Microbiol. 7:39. doi: 10.3389/fcimb.2017.00039 28261568 PMC5310132

[B28] Niu L. Gao M. Li Y. Wang C. Zhang C. Duan H. . (2025). Effects of the stress hormone norepinephrine on the probiotic properties of *Levilactobacillus*: antibacterial colonization, anti-inflammation, and antioxidation. Front. Microbiol. 16:1526362. doi: 10.3389/fmicb.2025.1526362 39996081 PMC11849050

[B29] Ovcharova M.A. Geraskina O.V. Danilova N.D. Botchkova E.A. Martyanov S.V. Feofanov A.V. . (2021). Atrial Natriuretic Peptide Affects Skin Commensal *Staphylococcus epidermidis* and *Cutibacterium acnes* Dual-Species Biofilms. Microorganisms 9:552. doi: 10.3390/microorganisms9030552 33800171 PMC7999105

[B30] Ovcharova M.A. Schelkunov M.I. Geras'kina O.V. Makarova N.E. Sukhacheva M.V. Martyanov S.V. . (2023). C-Type Natriuretic Peptide Acts as a Microorganism-Activated Regulator of the Skin Commensals *Staphylococcus epidermidis* and *Cutibacterium acnes* in Dual-Species Biofilms. Biology 12:436. doi: 10.3390/biology12030436 36979128 PMC10045295

[B31] Russo P. Iturria I. Mohedano M.L. Caggianiello G. Rainieri S. Fiocco D. . (2015). Zebrafish gut colonization by mCherry-labelled lactic acid bacteria. Appl. Microbiol. Biotechnol. 99:3479. doi: 10.1007/s00253-014-6351-x 25586576

[B32] Sarkodie E. K. Zhou S. Baidoo S. A. Chu W. (2019). Influences of stress hormones on microbial infections. Microb. Pathog. 131, 270–276. doi: 10.1016/j.micpath.2019.04.013 30981718

[B33] Vorregaard M. (2008). Comstat2 - a modern 3D image analysis environment for biofilms. undefined. Available online at: https://www.semanticscholar.org/paper/Comstat2-a-modern-3D-image-analysis-environment-for-Vorregaard/87105161b4e817b6d74bd0fbbbbb4edc52e30830 (Accessed June 8, 2022).

[B34] Zommiti M. Bouffartigues E. Maillot O. Barreau M. Szunerits S. Sebei K. . (2018). In vitro Assessment of the Probiotic Properties and Bacteriocinogenic Potential of *Pediococcus pentosaceus* MZF16 Isolated From Artisanal Tunisian Meat “Dried Ossban.” Front. Microbiol. 9:2607. doi: 10.3389/fmicb.2018.02607 30473681 PMC6238632

